# Tablet Acceptability in Older Outpatients Undergoing Cancer Chemotherapy

**DOI:** 10.3390/geriatrics11020025

**Published:** 2026-02-26

**Authors:** Eri Hikita, Mami Oosaki, Ayano Suzuki, Maiko Anzai, Nanako Yoshioka, Yoshiyasu Terayama, Takeo Yasu

**Affiliations:** 1 Department of Pharmacy, Tokyo Metropolitan Bokutoh Hospital, Tokyo 130-8575, Japan; eri_hikita@tmhp.jp (E.H.); mami_oosaki@tmhp.jp (M.O.); ayano_suzuki@tmhp.jp (A.S.); maiko_shimizu@tmhp.jp (M.A.); nanako_yoshioka@tmhp.jp (N.Y.); yoshiyasu_terayama@tmhp.jp (Y.T.); 2 Department of Medicinal Therapy Research, Pharmaceutical Education and Research Center, Meiji Pharmaceutical University, Tokyo 204-8588, Japan; 3 Bokutoh Hospital-Meiji Pharmaceutical University Joint Research Center, Tokyo 130-8575, Japan

**Keywords:** older outpatients, oral anticancer drugs, tablet acceptability, polypharmacy, oral frailty

## Abstract

**Background/Objectives:** Patient acceptability of oral anticancer drugs is a critical factor that influences treatment in older outpatients receiving cancer chemotherapy and plays a central role in enhancing adherence and treatment effectiveness. Identifying older outpatients receiving cancer chemotherapy who exhibit poor tablet acceptability before initiating oral anticancer therapy and offering alternative treatment options are beneficial. Therefore, we investigated the characteristics of patients with poor tablet acceptability by focusing on the tablet size, geriatric assessment, and polypharmacy. **Methods:** A questionnaire survey on experiences with tablet medication was conducted among patients who received chemotherapy at the Outpatient Treatment Center of Tokyo Metropolitan Bokutoh Hospital from September 2024 to September 2025. The median values of the long diameter (12 mm) and the combined length, width, and thickness (26 mm) of the tablets reported as acceptable in the questionnaire described in Method 1 were used as cutoff values. Patients whose reported acceptable tablet dimensions were below these median values were classified as “poor tablet acceptability,” whereas those with values above the median were classified as “good tablet acceptability”. Univariate and multivariate logistic regression analysis was performed to identify characteristic factors associated with poor tablet acceptability in older outpatients receiving cancer chemotherapy, with poor tablet acceptability as the dependent variable and patient sex, body mass index, Geriatric 8 score, each item of the Oral Frailty 5-item Checklist, and polypharmacy as explanatory variables. **Results:** 90 patients completed the questionnaire survey. Female sex and polypharmacy were independent factors associated with poor tablet acceptability in older outpatients receiving cancer chemotherapy. In addition, subjective difficulty in chewing tended to be associated with poor tablet acceptability. **Conclusions:** This study suggests that assessing polypharmacy and oral function, along with early multidisciplinary intervention before and during oral anticancer therapy, particularly in females, patients taking multiple medications, and those reporting difficulty in chewing, may help maintain tablet acceptability and improve adherence.

## 1. Introduction

Cancer is the second leading cause of death worldwide, accounting for 16.8% of all deaths [1]. The incidence of cancer in older adults is increasing rapidly, with approximately 30% of newly diagnosed patients with cancer in the United States aged 65–74 years and 25% aged ≥75 years [2]. More than 70% of cancer cases and >85% of cancer-related deaths occur in adults aged ≥65 years in Japan, one of the world’s most super-aged societies [3]. Advances in anticancer drugs and supportive care have established outpatient chemotherapy as the primary approach to cancer treatment, enabling patients to balance therapy with family and work commitments and continue treatment as part of their daily lives. Furthermore, the development of oral anticancer drugs is progressing rapidly, with a substantial number of approved and marketed agents. Treatment regimens incorporating oral anticancer drugs, such as capecitabine and oxaliplatin (CAPOX) regimen [4], have become the standard of care for several carcinomas. In addition, oral anticancer drugs offer high convenience and are expected to continue to be widely used in the future to improve the quality of life of patients. Patient tablet acceptability refers to an individual’s overall ability and willingness to take medications as prescribed [5] and is intricately related to medication adherence [6]. Oral anticancer drugs cannot achieve therapeutic effects if not taken consistently [7], making the acceptability of patient medicine a critical factor.

Cancer drug therapy for older adults faces challenges such as declining physical function, diverse comorbidities, cognitive limitations, polypharmacy, socioeconomic constraints, and substantial individual variability in these factors [8,9,10,11,12,13,14]. In older patients with cancer, ensuring that oral anticancer drugs are appropriately sized for individuals with diverse comorbidities is important for maintaining adherence. Tablet acceptability in older adults largely depends on the tablet size [15], and older patients with dysphagia can only tolerate tablets with a maximum long diameter of 6.5 mm [16]. Furthermore, in addition to the long diameter, when the combined length, width, and thickness of a tablet exceed 21 mm, adherence is negatively affected [17]. Both the long diameter and combined length, width, and thickness of oral anticancer drugs marketed in Japan have demonstrated an increasing trend over the successive years since their launch [18]. Furthermore, 84.2% of oral anticancer drugs marketed have a long diameter of ≥7 mm, and 56.8% have a combined length, width, and thickness of ≥21 mm, suggesting that the dosage form of oral anticancer drugs may negatively affect tablet acceptability in older patients with cancer [18].

Geriatric Assessment (GA) is a tool for comprehensively evaluating the physical, mental, and social functions of older patients with cancer [19]. The GA, which identifies patient vulnerabilities and may be useful for guiding treatment strategies and predicting chemotherapy-related adverse events, is recommended for use in clinical trials and routine practice worldwide [19]. The Geriatric 8 (G8), a simple screening tool, includes items related to older adult function, such as physical function, medications, nutritional status, and mood. [20] It has been reported to be the most useful because it has high sensitivity, acceptable specificity, robust data, and is a prognostic factor for many cancers [21,22]. In recent years, oral frailty (OF) has gained attention as a potential trigger for physical decline in older adults. The Oral Frailty 5-item Checklist (OF-5), which can be used to assess OF even without a dental professional, evaluates patients across five items: (1) subjective difficulty in chewing, (2) difficulty in swallowing, (3) dry mouth, (4) impaired speech, and (5) reduced number of remaining teeth. OF was defined as the presence of two or more of the five items [23]. It has been reported that OF may precede physical frailty [23] and that physical disability and mortality are increased in older individuals with OF [23].

Polypharmacy is one of the most important factors that contribute to poor medication adherence in older individuals [24]. In cancer treatment, polypharmacy similarly affects medication adherence [25]. Polypharmacy has been reported to contribute to poor adherence to tegafur/gimeracil/oteracil potassium combination drug (S-1) in patients with gastric cancer [26]. The number of drugs that constitute polypharmacy is unclear. In this study, polypharmacy is defined as six or more commonly used drugs. [27,28]

Patient tablet acceptability [5,6,29,30] for oral anticancer drugs is an important factor influencing the treatment of older outpatients with cancer receiving chemotherapy, and is crucial for improving adherence to oral anticancer drugs and overall treatment effectiveness. As noted above, identifying older outpatients with cancer receiving chemotherapy with poor tablet acceptability before initiating oral anticancer therapy and offering alternative treatment options would be beneficial. However, no studies have evaluated the relationship between tablet acceptability and GA or polypharmacy in older patients with cancer. Therefore, we examined the characteristics of older outpatients with cancer receiving chemotherapy with poor tablet acceptability, focusing on tablet size, GA, and polypharmacy to be aimed to propose appropriate cancer chemotherapy options for older outpatient cancer chemotherapy patients.

In this study, older adults are defined as those aged 65 years or older, based on previous studies [15,23,31] and guidelines [13].

## 2. Materials and Methods

### 2.1. Questionnaire Survey on Experiences with Tablet Medication in Older Outpatients with Cancer Receiving Chemotherapy

**Figure 1:**
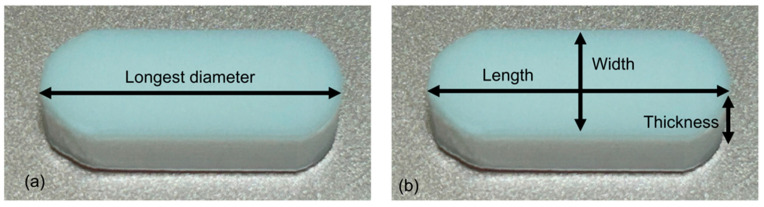
Definitions of (**a**) the longest diameter and (**b**) the combined length, width, and thickness of tablets.

A questionnaire survey on experiences with tablet medications (Table A1) was conducted among patients receiving chemotherapy at the Outpatient Treatment Center of Tokyo Metropolitan Bokutoh Hospital from September 2024 to September 2025. The survey was conducted by pharmacists through individual interviews after obtaining patient consent to participate in the study. The study included only adults who were deemed capable of providing informed consent; minors and adults for whom valid informed consent was considered difficult to obtain, such as those with dementia, were excluded. Written informed consent was obtained from all participants. Patient interviews were conducted by an experienced pharmacist holding specialist certification in oncology and having extensive experience in interviewing patients with cancer. The definitions of the longest diameter and combined length, width, and thickness of the tablets are shown in Figure 1. For Question 1, seven straight lines (7, 10, 12, 14, 16, 18, and 20 mm) representing tablet long diameters were drawn, ranging from 7 mm, which is considered the easiest diameter for older individuals to swallow, to 20 mm, which is the maximum long diameter of oral anticancer drugs marketed in Japan. The patients were asked to select the maximum long diameter that they deemed acceptable for the medication. For Question 2, the patients were presented with oval tablet models of up to 30 mm, representing the maximum combined length, width, and thickness of oral anticancer drugs marketed in Japan, including 21 mm, which has been reported to affect medication adherence [8]. As in Question 1, the patients were asked to select the largest tablet model that they deemed acceptable for medication. Aligned with Question 1, seven tablet models (No. 1–7) were presented for selection based on their longest diameter (Table 1). Question 3 investigated the maximum number of tablets that patients believed they could take at one time based on the tablet size they indicated as acceptable in the previous questions. Questions 4 to 9 focused on the G8 assessment to assess physical function. Administered by healthcare professionals, and with a score of ≤14 out of 17 indicated a potential decline in physical or other functions. Age and body mass index (BMI) data were obtained from medical records. Questions 10–14 addressed the OF-5. Swallowing function was assessed by patient self-report. Patients were asked to report all medications they were regularly taking at that time.

### 2.2. Comparisons of G8 and OF-5 Scores Between Patients with Poor vs. Good Tablet Acceptability in Older Outpatients with Cancer Receiving Chemotherapy

In this study, given the unique target population—older outpatients with cancer on oral anticancer agents—we pre-specified cohort medians of the maximum acceptable long diameter (12 mm) and the combined length, width, and thickness (26 mm) as practice-anchored thresholds for poor acceptability, rather than adopting literature-based thresholds derived from non-cancer or mixed-medication cohorts.

Accordingly, tablet acceptability was defined using these median-based thresholds obtained from the questionnaire described in Method 1. Patients whose self-reported maximum acceptable tablet dimensions were below either median value were classified as having “poor tablet acceptability,” whereas those with values at or above the median were categorized as having “good tablet acceptability” [28]. We compared G8 and OF-5 scores between patients classified as having poor versus good tablet acceptability, as defined by the pre-specified cohort medians (long diameter 12 mm; the combined length, width, and thickness 26 mm).

### 2.3. Investigation of Characteristic Factors in Older Outpatients with Cancer Receiving Chemotherapy with Poor Tablet Acceptability

To explore factors associated with tablet acceptability, we evaluated patient sex (male or female), each item of the Oral Frailty 5-item Checklist (presence or absence of difficulty in chewing, dysphagia, dry mouth, impaired speech, and number of remaining teeth [≥20 or ≤19]), and the presence or absence of polypharmacy, treating all variables as nominal.

### 2.4. Questionnaire Survey for Healthcare Professionals on Drug Adherence

We have reported that both the long diameter and the combined length, width, and thickness of oral anticancer drugs marketed in Japan have increased over successive years since their launch, and that the dosage form of oral anticancer drugs itself may reduce patient medication adherence [18]. However, few healthcare professionals were aware of these findings. Therefore, we considered the possibility of a discrepancy between the tablet size that older patients with cancer believe they can swallow and the tablet size that healthcare professionals, including prescribing physicians, perceive as manageable and the possibility that oral anticancer drugs were being prescribed even though the patient was unable to take them. A questionnaire survey was conducted by pharmacists using questions 1 and 2 of method 1 to evaluate the perceptions of healthcare professionals and patients or among healthcare professionals regarding tablet acceptability. After obtaining consent from healthcare professionals (physicians, nurses, and pharmacists), similar to the process for patients, participants were asked to indicate the long diameter and the combined length, width, and thickness of tablets that they believed their patients could safely take.

### 2.5. Statistical Analysis

We compared G8 scores and OF-5 scores between the poor and good acceptability groups using the Mann–Whitney U test (two-sided). For comparisons among the four groups (patients, physicians, nurses, and pharmacists), we used the Kruskal–Wallis test to assess overall differences in the maximum tablet size considered acceptable. When the overall test was significant, we performed post hoc multiple comparisons using the Steel–Dwass method. Logistic regression analysis was performed to identify the characteristic factors associated with poor tablet acceptability. The dependent variable was poor tablet acceptability, and the explanatory variables included patient sex (male or female), each item of the OF-5 (presence or absence of difficulty in chewing, dysphagia, dry mouth, impaired speech, and number of remaining teeth [≥20 or ≤19]), and presence or absence of polypharmacy. Univariate analysis was performed, followed by multivariate logistic regression using explanatory variables with a *p*-value < 0.05. Odds ratios (OR) and 95% confidence intervals (95% CI) were calculated, with *p* < 0.05 considered statistically significant. All statistical analyses were performed using the EZR software [32]. EZR is a statistical software package that extends the functionality of R and its commands. The study was conducted in accordance with the principles of the Declaration of Helsinki and approved by the Ethics Committee of Meiji Pharmaceutical University (No. 202412, 202413) and the Tokyo Metropolitan Bokutoh Hospital (approval number: 06-006).

## 3. Results

### 3.1. Questionnaire Survey on Experiences with Tablet Medication in Older Outpatients with Cancer Receiving Chemotherapy

**Figure 2:**
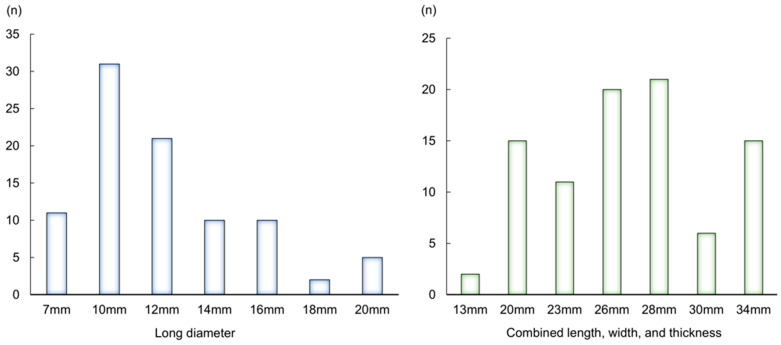
The long diameter and the combined length, width, and thickness of tablets which were reported as acceptable by patients.

A questionnaire was administered to 90 patients. The median age was 75 (range: 65–89) years, with 51 males and 39 females in total. The types of cancer included lung cancer in 26 patients, colorectal cancer in 14, pancreatic cancer in 12, hematologic cancers in 12 (leukemia in 1, lymphoma in 6, and multiple myeloma in 5), breast cancer in 10, gastric cancer in 5, hepatocellular carcinoma in 5, ovarian cancer in 2, biliary tract cancer in 2, uterine cancer in 1, and endocrine tumors in 1 patient. A potential decline in physical function, indicated by a G8 score of ≤14 points, was observed in 71 patients (78.9%), and OF, defined by an OF-5 score of ≥2 points, was present in 49 patients (54.4%). The median number of regular medications among the patients was six (range: 0–13), and 55 patients (61%) exhibited polypharmacy (Table 2). The median long diameter of the tablets reported as acceptable by the patients was 12 mm, and the median combined length, width, and thickness was 26 mm (Figure 2). A maximum long diameter of ≥12 mm for regular medications was reported by 26 patients (29%), and a maximum combined length, width, and thickness of ≥26 mm was reported in 27 patients (30%) (Table 2). The patients reported that they could take a median of two tablets at a time when the combined length, width, and thickness was 26 mm.

### 3.2. Comparisons of G8 and OF-5 Scores Between Patients with Poor vs. Good Tablet Acceptability in Older Outpatients with Cancer Receiving Chemotherapy

Among 90 elderly outpatients, the median G8 and OF-5 scores were 13.0 and 1, respectively. Under the long diameter, G8 score showed a borderline difference between groups (median 13.75 in the poor group vs 13.00 in the good group, *p* = 0.053). In contrast, OF-5 scores did not differ between groups under the long diameter. When tablet acceptability was defined by the combined length, width, and thickness, neither G8 nor OF-5 scores differed significantly between the poor and good groups.

### 3.3. Exploration of Characteristic Factors in Older Outpatients with Cancer Receiving Chemotherapy with Poor Tablet Acceptability

The univariate logistic regression analysis revealed that female sex and polypharmacy were associated with a *p*-value < 0.05, and subjective difficulty in chewing was associated with a *p*-value < 0.1 (OR = 2.410, 95% CI: 0.921–6.29, *p* = 0.073) for the long diameter. For the combined length, width, and thickness, only subjective difficulty in chewing was associated with a *p*-value < 0.1 (OR = 2.440, 95% CI: 0.921–6.460, *p* = 0.073). Multivariate logistic regression analysis was performed using the two variables with a *p*-value < 0.05 for the long diameter. The results demonstrated that female sex (OR = 2.420, 95% CI: 1.000–5.830, *p* = 0.049) and polypharmacy (OR = 2.820, 95% CI: 1.130–7.000, *p* = 0.026) were independent factors. Subjective difficulty chewing tended to be a characteristic factor in patients with poor tablet acceptability (Table 3).

### 3.4. Questionnaire Survey for Healthcare Professionals on Drug Adherence

**Figure 3:**
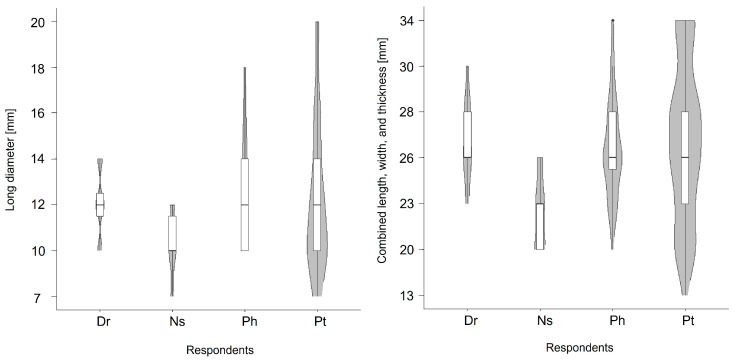
The long diameter and the combined length, width, and thickness of tablets that healthcare professionals believed older cancer patients could take. Dr: doctor, Ns: nurse, Ph: pharmacist, Pt: patient.

A questionnaire was administered to 54 healthcare professionals. The participants consisted of 12 physicians, 14 nurses, and 28 pharmacists. The median values for the long diameter and combined length, width, and thickness of tablets that healthcare professionals believed older cancer patients could take were 12 mm (range: 10–14 mm) and 26 mm (range: 23–30 mm) by physicians; 10 mm (range: 7–12 mm) and 23 mm (range: 20–26 mm) by nurses; and 12 mm (range: 10–18 mm) and 26 mm (range: 20–34 mm)by pharmacists (Figure 3). The long diameter and combined length, width, and thickness of tablets that older patients with cancer were considered capable of taking were the smallest according to nurses and the largest according to pharmacists.

In the four-group comparison (patients, physicians, nurses, and pharmacists), the Kruskal–Wallis test indicated significant overall differences for both the long diameter and the combined length, width, and thickness considered acceptable tablets (each *p* < 0.05). In Steel–Dwass post hoc analyses for the long diameter, nurses selected significantly smaller sizes than physicians (*p* = 0.0342) and pharmacists (*p* = 0.0079). Similarly, for the combined length, width, and thickness of tablets, nurses selected significantly smaller sizes than physicians (*p* = 0.0028) and pharmacists (*p* = 0.0011). In addition, nurses showed significantly smaller combined length, width, and thickness of tablets than patients *(p* = 0.0043).

## 4. Discussion

We investigated tablet acceptability in older outpatients with cancer receiving chemotherapy and found that the maximum tablet size deemed acceptable had a median long diameter of 12 mm and a median combined length, width, and thickness of 26 mm. In addition, multivariate analysis revealed that female sex and polypharmacy were independent factors associated with poor tablet acceptability. Furthermore, patients who reported subjective difficulty chewing tended to exhibit poor tablet acceptability. This study is novel in that it is the first to examine the independent characteristic factors associated with poor tablet acceptability in older outpatients with cancer receiving chemotherapy, considering physical function assessments (G8 and OF-5) and polypharmacy.

The older outpatients with cancer reported that they could accept tablet sizes larger than previously reported as suitable for older individuals, 6.5 mm for the long diameter [16] and 21 mm for the combined length, width, and thickness, which has been reported to affect medication [17]. Although direct comparison with the populations in previous reports is not possible, we found that despite the majority (78.9%) of older outpatients with cancer receiving chemotherapy in this study having a potential decline in physical function, indicated by a G8 score of ≤14, they were physically fit enough to undergo chemotherapy as outpatients. Therefore, differences in the study populations may have influenced the results. In addition, the discrepancy between the results for the long diameter and the combined length, width, and thickness may reflect differences in the nature of these two measures; the long diameter is possibly based primarily on visual assessment, whereas the total diameter requires a three-dimensional evaluation. This may also be influenced by age-related decline in patients’ visual function [33,34] and individual variability [35].

When tablets are large and difficult to swallow, crushed preparations or administration using a simple suspension method are sometimes employed in clinical practice in Japan [36]. However, elevated blood drug concentrations have been reported when oral anticancer drugs are administered in crushed form [37]. To date, only a few studies have evaluated the effectiveness or safety of oral anticancer drug suspensions [38], and information on the suspension method remains limited [18,39,40]. Regarding the use of crushed preparations and simple suspension administration of anticancer drugs, the risk of drug exposure to healthcare professionals and caregivers must also be considered [41,42,43].

Capecitabine is indicated for the treatment of colorectal and breast cancers [44]. Combination therapy (with the CAPOX regimen) serves as a postoperative adjuvant therapy for colorectal cancer and as a first-line treatment for advanced colorectal and gastric cancers [45]. The long diameter of capecitabine was near the cutoff value for tablet acceptability identified in this study (12 mm), raising concerns that treatment continuation may be challenging for patients with poor tablet acceptability. If capecitabine is unacceptable, tegafur/gimeracil/oteracil (TS-1) has been approved in Asia, and equivalence between CAPOX and SOX regimens for gastric cancer has been reported [46,47]. Thus, the SOX regimen, which uses TS-1 with a smaller tablet size, could serve as an alternative; however, it is not recommended for colorectal cancer. Furthermore, the CAPOX regimen has been demonstrated to improve outcomes in patients with colorectal cancer compared to the FOLFOX regimen, in which the fluoropyrimidine anticancer drug fluorouracil is administered intravenously instead of capecitabine. Therefore, switching to the CAPOX regimen is not recommended without careful consideration [48]. In addition, a significant difference in overall survival was observed between older cancer patients who discontinued capecitabine early and those who completed postoperative adjuvant chemotherapy for colorectal cancer [49]. Furthermore, adding oxaliplatin to capecitabine monotherapy (the CAPOX regimen) is ineffective in older patients with cancer [50]. These reports indicate that capecitabine is a key drug in the treatment of colorectal cancer, and poor patient tablet acceptability is a critical challenge that directly affects treatment effectiveness.

Osimertinib requires long-term administration for recurrent or refractory lung cancer as well as for postoperative adjuvant chemotherapy. Osimertinib as postoperative adjuvant chemotherapy significantly prolongs disease-free survival, even in older patients with cancer [51]. The problem is that Osimertinib is combined length, width, and thickness of ≥ 26 mm. However, if patients are unable to take osimertinib, substitution with other tyrosine kinase inhibitors is not feasible due to the lack of approved indications. Gefitinib and erlotinib are alternative options for advanced cancers; however, the combined length, width, and thickness of gefitinib and erlotinib tablets are 27.4 mm and 26.4 mm, respectively. Similar to osimertinib, these dimensions exceeded the median acceptable limit of 26 mm, making ingestion difficult for patients with poor tablet acceptability. When tyrosine kinase inhibitors cannot be administered, cytotoxic anticancer drugs are the treatment of choice [45]; however, they substantially increase the physical burden on patients.

To provide effective and safe cancer treatment for patients with poor tablet acceptability, it is desirable to introduce tablets with smaller dimensions, including lower-dose formulations, and develop orally disintegrating tablets using advanced formulation technologies.

As of 2023, both the long diameter and combined length, width, and thickness of oral anticancer drugs prescribed in Japan have demonstrated an increasing trend annually since their launch [18]. We hypothesized that a few healthcare professionals were aware that the dosage of oral anticancer drugs could reduce patient acceptance of medication. This study is the first to report differences in perceptions of patient tablet acceptability across professions in terms of tablet size considered acceptable by older patients with cancer, as perceived by prescribing physicians, pharmacists, and nurses who interact with patients. The median tablet size that prescribing physicians perceived as acceptable for older patients with cancer was consistent with the median tablet size reported by the patients, suggesting that physicians may have a general understanding of the tablet size that is compatible with their patients’ tablet acceptability. However, the consistency in median values does not indicate whether individualized considerations were adequately applied to approximately 30% of the patients with poor tablet acceptability. The median values perceived by nurses, who frequently administer medications to older patients, suggest that they may have considered patients’ swallowing function and associated medication risks. Unlike nurses, pharmacists have limited opportunities to be directly involved in medication administration. The manner of involvement in medication administration to older patients may have contributed to the differences in the perceptions of patient tablet acceptability between professions. The results of this study suggest that multidisciplinary discussions are significant in addressing the acceptability of oral anticancer drugs among older patients with cancer. In contrast, the number of years of professional experience may also influence perceptions of tablet acceptability; therefore, further investigation is warranted.

In this study, we conducted oral functions of older outpatients receiving cancer chemotherapy and found that 54.4% exhibited OF, as determined using the OF-5 screening tool. A study of patients receiving cancer chemotherapy found a higher incidence of OF among these patients (57.5%) than among older individuals in the community (43.6%) [52]. Subjective difficulty in chewing, an item on the OF-5, tended to be a characteristic of older outpatients receiving cancer chemotherapy who exhibited poor tablet acceptability.

Although chewing and swallowing are often considered single functions, they are independent components of oral performance. The self-assessment of chewing may reflect oral dysfunction and is intricately associated with oral conditions, oral complaints, and self-assessed health [53]. In addition, when difficulty in chewing and dysphagia were examined as independent predictors of frailty, subjective difficulty in chewing was significantly associated with frailty in older individuals [54]. Older outpatients receiving cancer chemotherapy who experience subjective difficulty chewing may be unable to accept oral anticancer drugs with large tablet sizes, potentially reducing the therapeutic efficacy of chemotherapy. It is important for healthcare professionals involved in outpatient cancer chemotherapy to closely monitor patients reporting difficulty in chewing and implement measures, such as chewing training with gum [55] and interventions to slow sarcopenia progression [56], to preserve chewing function and maintain the acceptability of oral anticancer drugs.

In older individuals, sex differences in chewing function have been suggested [57], and in the present study, female sex was associated with characteristics of patients exhibiting poor tablet acceptability. Moreover, sex differences exist in the factors influencing chewing ability in older individuals [58], and the effect of oral factors, such as the number of remaining teeth, occlusal force, and oral health, on chewing ability tends to vary by sex [59]. Sex differences in oral function, as well as psychological and cultural factors, may explain why female sex was identified as an independent factor associated with poor tablet acceptability in this study, although further research is needed. Regular evaluation and maintenance of oral conditions using the OF-5, which assesses a broad range of oral functions, including chewing, number of remaining teeth, and articulation, before and during oral anticancer drug therapy, may help ensure acceptability and adherence to oral anticancer drugs in older outpatients receiving cancer chemotherapy. Collaboration with other healthcare professionals, such as dentists, is important in these circumstances. Consequently, we plan to conduct clinical studies to evaluate the relationship among OF-5 assessments, multidisciplinary interventions, and medication adherence.

Polypharmacy has been reported as a factor that contributes to poor medication adherence [25] and is associated with reduced compliance with oral anticancer drugs [26]. Multivariate analysis revealed for the first time that polypharmacy, defined as six or more regular medications, is an independent characteristic in older outpatients receiving cancer chemotherapy who exhibit poor tablet acceptability. As in the rest of the world, polypharmacy represents a major concern in Japan, which is a rapidly aging society [60]. In particular, patients with cancer are prone to taking multiple medications, including those for comorbidities, supportive care, symptom management, and analgesia, during cancer treatment, and 88.4% of older individuals with cancer are affected by polypharmacy [61]. In this study, 61% of the older outpatients receiving cancer chemotherapy were taking six or more medications, highlighting the high prevalence of polypharmacy in this population. Optimizing polypharmacy by streamlining and reducing regular medications may help prevent a decline in tablet acceptability and support adherence to oral anticancer drugs in older outpatients receiving cancer chemotherapy. To prevent polypharmacy in older outpatients receiving cancer chemotherapy who often visit multiple medical institutions, it is important to enhance information sharing and strengthen collaboration among hospitals providing cancer treatment, patients’ regular healthcare providers, and insurance pharmacies. Anticholinergic medications are associated with the development of OF symptoms such as dry mouth and dysphagia [54,62]. Anticholinergic medication has also been reported to be associated with an increased risk of frailty [63] and cognitive impairment [64]. Regular prescriptions often include multiple anticholinergic medications in patients undergoing polypharmacy. Anticholinergic drugs may negatively affect dry mouth and swallowing function, thereby reducing their acceptability. Patient tablet acceptability, and medication adherence should be evaluated to address polypharmacy, the relationships among anticholinergic use.

This study had several limitations. First, in this study, we conducted a survey involving patients with a wide variety of cancer types and were able to evaluate average outcomes across the overall cancer population. However, as shown in Table 2, patients with head and neck cancer were not included in this study. Therefore, the findings of this study cannot be extrapolated to patients with head and neck cancer or esophageal cancer, who often experience swallowing dysfunction as a result of cancer treatments such as surgery or radiotherapy. Second, the findings of this study were limited to outpatients, and hospitalized patients were excluded. Older outpatients undergoing cancer chemotherapy generally maintain a performance status that allows them to attend hospital visits; however, hospitalized patients may have a lower performance status and more severe physical conditions compared with the study population. Therefore, the results obtained in this study cannot be generalized to hospitalized patients. Third, this study did not perform a prior sample size calculation, so the power of the study may have been insufficient, and the results should be interpreted with caution. Fourth, this study focused on older patients with cancer at a single institution in Japan, highlighting the need for further research involving a broader patient population. Differences in oral functions, such as chewing, between racial groups [65] may limit the generalizability of the results of this study to other countries. Fifth, tablet acceptability was assessed via patient self-reporting using tablet models rather than actual tablet administration; therefore, the results may not fully reflect the acceptability of real medications. Furthermore, the results based on healthcare professionals’ perceptions of tablet acceptability among older cancer patients may not sufficiently reflect individual patient needs, and further studies are needed to confirm generalizability. In future studies, we also plan to collect and analyze long-term follow-up data to evaluate whether low acceptability predicts actual non-adherence.

## 5. Conclusions

This study elucidated the current status of tablet acceptability in older outpatients receiving cancer chemotherapy and, for the first time, used multivariate analysis to demonstrate that female sex and polypharmacy (six or more regular medications) were independent factors associated with poor tablet acceptability. In addition, this study indicated that subjective difficulty in chewing tended to be associated with reduced tablet acceptability. These findings suggest that assessing polypharmacy and oral function, along with early multidisciplinary intervention before and during oral anticancer drug therapy, particularly in female patients, those taking multiple medications, and those reporting difficulty in chewing, may help preserve tablet acceptability and enhance adherence.

## Figures and Tables

**Table 1:** Total [mm], long diameter [mm], short diameter [mm], and thickness [mm] of seven tablet models.

Tablet Model (No.)	1	2	3	4	5	6	7
Total [mm]	13	20	23	26	28	30	34
Long diameter [mm]	7	10	12	14	16	18	20
Short diameter [mm]	4	6	7	7	7	7	8
Thickness [mm]	2	4	4	5	5	5	6

**Table 2:** Characteristics of study patients. Continuous variables are expressed as median (range) as appropriate. Categorical variables are presented as a number (%).

Characteristics	Value (*n* = 90)
**Sex, male/female**	51/39
**Age, years**	75 [65–89]
**Body Mass Index** **(** **BMI** **)** **, kg/m^2^**	22.5 [15.57–35.64]
**Geriatric-8 score ≤ 14 (problem)**	71
**Oral frailty (yes)**	49
**Number of regularly taken drugs median**	6 [0–13]
**Polypharmacy** **(** **≥6 drugs** **)**	55
**Longest diameter of regularly taken drugs ≥ 12 mm**	26
**Combined length, width, and thickness of regularly taken drugs ≥ 26 mm**	27
**Cancer type**	
**Lung cancer**	26
**Colorectal cancer**	14
**Pancreatic cancer**	12
**Hematological malignancies**	12
**Breast cancer**	10
**Gastric cancer**	5
**Hepatocellular carcinoma**	5
**Ovarian cancer**	2
**Biliary tract cancer**	2
**Uterine cancer**	1
**Endocrine tumors**	1

**Table 3:** Exploration of characteristic factors in older outpatients with cancer receiving chemotherapy with poor tablet acceptability.

	Longest Diameter				Combined Length, Width, and Thickness	
Variable	Univariate OR (95% CI)	Univariate *p*-Value	Multivariate OR (95%CI)	Multivariate *p*-Value	Univariate OR (95% CI)	Univariate *p*-Value
Sex female	2.420 (1.030–5.69)	0.042	2.420 (1.000–5.830)	0.049	1.200 (0.489–2.950)	0.691
Difficulty chewing (yes)	2.410 (0.921–6.29)	0.073			2.440 (0.921–6.460)	0.073
Difficulty swallowing (yes)	2.590 (0.719–9.32)	0.146	-	-	2.550 (0.741–8.750)	0.138
Dry mouth (yes)	1.140 (0.493–2.66)	0.753	-	-	1.370 (0.557–3.380)	0.491
Speech disorder (yes)	0.633 (0.233–1.72)	0.371	-	-	1.140 (0.403–3.240)	0.802
Reduction in number of remaining teeth <20	1.230 (0.533–2.82)	0.631	-	-	0.950 (0.388–2.330)	0.911
Polypharmacy (yes)	2.820 (1.160–6.870)	0.023	2.820 (1.130–7.000)	0.026	1.22 (0.48–3.070)	0.678

## Data Availability

The authors confirm that the data supporting the findings of this study are available within the article.

## Abbreviations

The following abbreviations are used in this manuscript:

BMI	Body mass index
CAPOX	Capecitabine and oxaliplatin
CI	Confidence interval
GA	Geriatric Assessment
OF	Oral frailty
OR	Odds ratios

## Appendix A

**Table A1:** Questionnaire survey on experiences with tablet medication.

Q1, If the size of the medication corresponds to the lines below, up to which number do you think you could take? Q2, Please refer to these tablet models. Up to which numbered medication do you think you could take? Q3, Of the tablets you indicated in Q2, how many do you think you could take at one time? Q4, Has food intake declined over the past 3 months due to loss of appetite, digestive problems, chewing, or swallowing difficulties? 1. Severe decrease in food intake 2. Moderate decrease in food intake 3. No decrease in food intake Q5, Weight loss during the last 3 months? 1. Weight loss > 3 Kg 2. Does not know 3. Weight loss between 1 and 3 kg 4. No weight loss Q6, Mobility? 1. Goes out 2. Able to get out of bed/chair but does not go out 3. Bed or chair-bound Q7, Neuropsychological problems? 1. Severe dementia or depression 2. Mild dementia 3. No psychological problems Q8, Take more than 4 prescription drugs per day? 1. Yes 2. No Q9, In comparison with other people of the same age, how does the patient consider his/her health status? 1. Not as good 2. Does not know 3. As good 4. Better Q10, Do you have difficulty eating hard foods compared to six months ago? 1. Yes 2. No Q11, Do you sometimes choke on tea, soup, etc.? 1. Yes 2. No Q12, Do you feel dry mouth? 1. Yes 2. No Q13, Do you have trouble pronouncing words clearly in everyday conversation? 1. Yes 2. No Q14, Count by dentist 1. 0~19 2. ≧20
